# Identification and comparative profiling of miRNAs in herbaceous peony (*Paeonia lactiflora* Pall.) with red/yellow bicoloured flowers

**DOI:** 10.1038/srep44926

**Published:** 2017-03-20

**Authors:** Daqiu Zhao, Mengran Wei, Min Shi, Zhaojun Hao, Jun Tao

**Affiliations:** 1 Jiangsu Key Laboratory of Crop Genetics and Physiology, College of Horticulture and Plant Protection, Yangzhou University, Yangzhou 225009, P.R. China

## Abstract

Herbaceous peony (*Paeonia lactiflora* Pall.) is popular worldwide because of its gorgeous flower colour, and the yellow flower is the rarest. However, its mechanism of yellow formation is still unexplored from the post-translational level. In this study, the anatomy of the petal, cell sap pH and metal elements were investigated in bicoloured flower cultivar ‘Jinhui’ with red outer-petal and yellow inner-petal, and the yellow formation was influenced by the anatomy of petal, while not by the cell sap pH and metal elements. Subsequently, microRNAs sequencing (miRNA-seq) was used to identify small RNAs (sRNAs). A total of 4,172,810 and 3,565,152 specific unique sRNAs were obtained, 207 and 204 conserved miRNAs and 38 and 42 novel miRNAs were identified from red outer-petal and yellow inner-petal, respectively, which were confirmed by subcloning. Among these miRNAs, 163 conserved and 28 novel miRNAs were differentially expressed in two wheel of petals. And 5 differentially expressed miRNAs and their corresponding target genes related to yellow formation were screened, and their dynamic expression patterns confirmed that the yellow formation might be under the regulation of miR156e-3p-targeted squamosa promoter binding protein-like gene (*SPL1*). These results improve the understanding of miRNA regulation of the yellow formation in *P. lactiflora*.

Herbaceous peony (*Paeonia lactiflora* Pall.) belonging to the Paeoniaceae family is fabulously elegant and gorgeous as tree peony (*Paeonia Suffruticosa* Andr.) as well as the king of herbaceous flowers, which implies the meaning of auspiciousness and wealth. *P. lactiflora* is rich in flower colours, which can be divided into nine categories including white, pink, red, purple, black, blue, yellow, green and double colours^1^. In the Chinese traditional culture, yellow is the colour reflecting wealth, and the price of *P. lactiflora* with yellow flowrs is as high as that of *P. Suffruticosa*, which is usually 10 times higher than that of red and purple *P. lactiflora*. And over 600 *P. lactiflora* cultivars, only one cultivar is pure yellow^2^, and the current market demand cannot be met. Therefore, clarifying the mechanism of yellow formation is crucial for breeding yellow *P. lactiflora* cultivars.

Flower colour is mainly decided by pigment, cell sap pH, metal elements, etc.^3^, and the yellow petals are mainly related to flavonoids and carotenoids^4^. For example, the cytoplasmic accumulation of flavonol glycosides was relevant to yellow flower colouration in lisianthus (*Eustoma grandiflorum* L.)^5^, and the yellowish sandersonia (*Sandersonia aurantiaca* Hook.) is due to the accumulation of the carotenoid pigments zeaxanthin and β-cryptoxanthin^6^. While in *P. lactiflora*, Jia *et al*.^7^ found that a large number of chalcone and a small amount of flavone and flavonol attributed to the yellow formation. And our previous studies also confirmed the above results using high-performance liquid chromatograph (HPLC) analysis of purplish-red, white and yellow *P. lactiflora* flowers^8^. On the molecular level, Jin *et al*.^9^ found that the mutation of chalcone isomerase gene (*CHI1/2*) coding regions abolished anthocyanin formation in the yellow cineraria (*Senecio cruentus* DC.) cultivar, and Zhang *et al*.^10^ found flavonoid 3′-hydroxylase gene (*F3*′*H*), dihydroflavonol 4-reductase gene (*DFR*) and anthocyanidin synthase gene (*ANS*) were not expressed in the yellow ornamental sunflower. In *P. lactiflora*, we isolated nine flavonoid biosynthetic genes and found that the low expression level of *CHI* induced most of the substrate accumulation in the form of chalcones and displaying yellow^8^. Furthermore, a bicoloured flower cultivar ‘Jinhui’ with a consistent genetic background red outer-petal and yellow inner-petal was used for transcriptome sequencing (RNA-Seq), and the coordinated expression of flavonoid biosynthetic genes mediated yellow formation in *P. lactiflora*, especially phenylalanine ammonialyase gene (*PAL*), flavonol synthase gene (*FLS*), *DFR, ANS*, flavonoid 3-*O*-glucosyltransferase gene (*F3GT*) and flavonoid 5-*O*-glucosyltransferase gene (*F5GT*)^11^. These findings clarifying the mechanism of yellow formation were all on the biochemical and transcription levels. However, the complex post-transcriptional regulation mechanism is equally important. Xu *et al*.^12^ found that microRNAs (miRNAs) had a functional role in the red colour formation of sweet orange flesh using miRNAs sequencing (miRNA-seq), which provided the reference for clarifying the mechanism of yellow formation from post-transcriptional level.

As a kind of small and endogenous non-coding RNAs, miRNAs play pivotal roles in post-transcriptional regulation of plant growth and development as well as stress responses^13^. Recently, with the rapid development of high-throughput sequencing technology, miRNA-seq has been widely applied in many plants, such as wheat (*Triticum aestivum* L.)^14^, trifoliate orange (*Poncirus trifoliata* L.)^15^, safflower (*Carthamus tinctorius* L.)^16^, peach (*Prunus persica* L. Batsch)^17^, Chinese white poplar (*Populus tomentosa* Carr.)^18^ and so on. And in ornamental flowers, rose (*Rosa* spp.)^19^, chrysanthemum (*Dendranthema morifolium* (Ramat.) Tzvel.)^20^, *P. lactiflora*^21^, Canna (*Canna indica* L.)^22^, etc. also have been reported in this application. Until now, 28,645 entries of hairpin precursor miRNAs (pre-miRNAs) were included in miRBase 21.0, which expressed 35,828 mature miRNA products over a range of 223 species^23^. Meanwhile, miRNAs have been found to be involved in numerous biological processes, such as signal conduction of hormones^24^,^25^, flower development^26^, vegetative organs development^27^,^28^, and defense responses against abiotic and biotic stresses^29^. With regard to miRNAs regulating colour formation, only a few studies have been reported to the best of our knowledge. Roy *et al*.^22^ used pale yellow and red flowers of *C. indica* to perform high-throughput sequencing, and found 109 differentially expressed miRNAs, 1,343 corresponding target genes gave 633 GO terms, among them, five miRNA families targeting five genes were involved in phenylpropanoid and pigment metabolic processes. However, no related studies of miRNAs regulating *P. lactiflora* colour formation have been reported.

In order to clarify the post-transcriptional regulation mechanism of yellow formation, a bicoloured flower cultivar ‘Jinhui’ with a consistent genetic background red outer-petal and yellow inner-petal including four developmental stages—flower-bud stage (Stage 1, S1), initiating bloom stage (Stage 2, S2), bloom stage (Stage 3, S3) and wither stage (Stage 4, S4)—was selected. The assessments included measurement of flower colour indices, observation of the anatomy of petals, determining cell sap pH, metal elements and organic acid contents, identifying differentially expressed miRNAs by miRNA-seq and analysing the dynamic expression patterns of candidate miRNAs and their corresponding target genes using real-time quantitative polymerase chain reaction (Q-PCR), thereby revealing the role of *P. lactiflora* miRNAs in the yellow formation. Taken together, these results would provide a foundation for breeding yellow *P. lactiflora* cultivars.

## Results

### Colour indices

**Figure 1:**
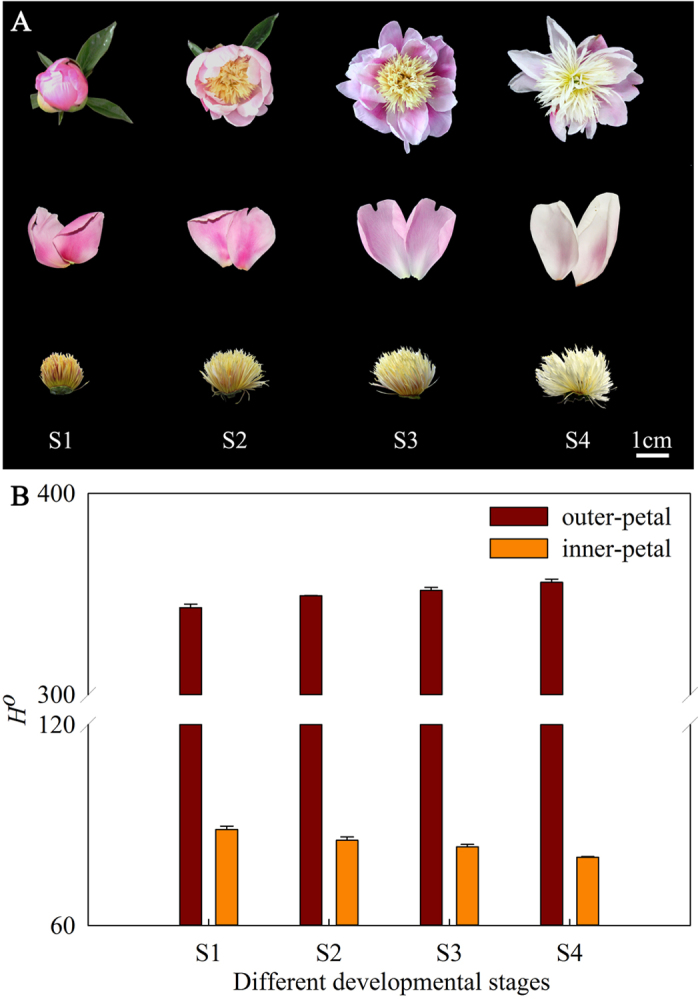
Photographs (**A**) and colour indices (**B**) of ‘Jinhui’ with red outer-petal and yellow inner-petal during the developmental stages. S1: Stage 1, flower-bud stage; S2: Stage 2, initiating bloom; S3: Stage 3, bloom stage; S4: Stage 4, wither stage.

To clarify the mechanism of yellow formation in *P. lactiflora*, a bicoloured flower cultivar ‘Jinhui’ with red outer-petal and yellow inner-petal was used as the experimental materials (Fig. 1A). Firstly, their colour indices were measured. In this study, the colour was represented by the hue angle (*H°*), which was as follows: 0° (360°) for reddish-purple, 90° for yellow, 180° for bluish-green and 270° for blue^30^. During the flower development, *H*° of red outer-petal gradually increased with 12.51°, while *H*° of yellow inner-petal constantly decreased with 8.30° (Fig. 1B). These results were in agreement with the petal colours of ‘Jinhui’ during the developmental stages, which indicated that the colours of the outer-petal and inner-petal were reddish-purple and yellow in S1, and then faded gradually as flower development proceeded.

### Anatomical structures

**Figure 2:**
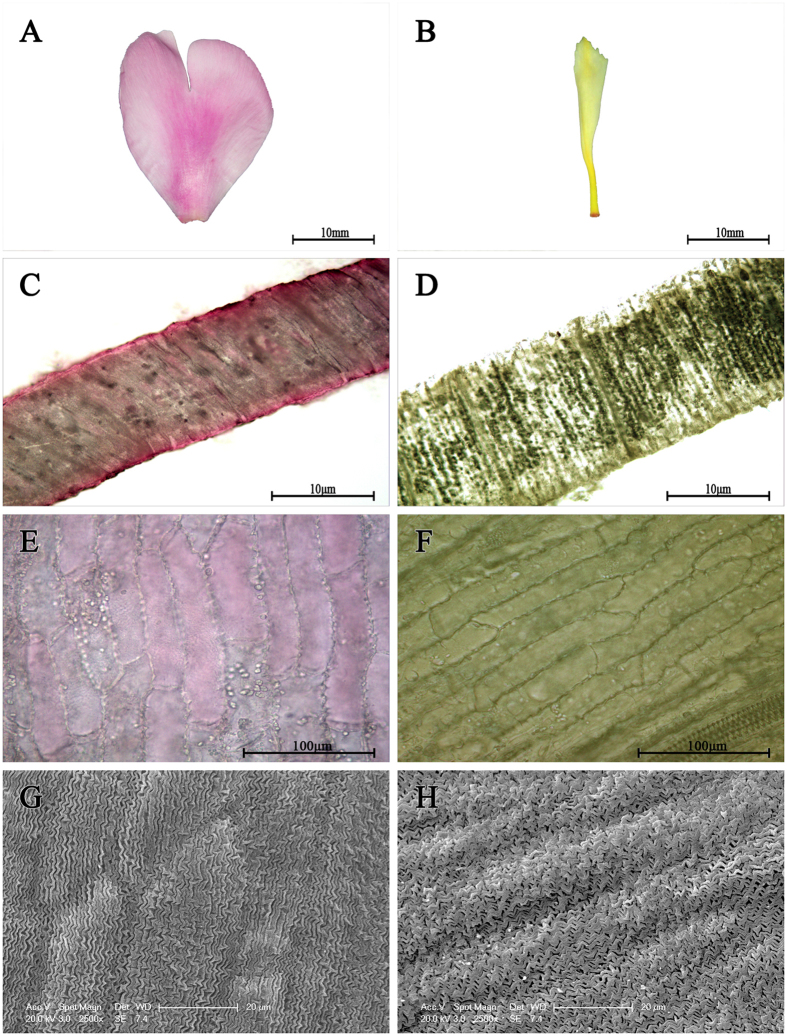
Cellular features of red outer-petal and yellow inner-petal. (**A**) Single red outer-petal. (**B**) Single yellow inner-petal. (**C**) Cross section of red outer-petal. (**D**) Cross section of yellow inner-petal. (**E**) Epidermal cells of red outer-petal. (**F**) Epidermal cells of yellow inner-petal. (**G**) Scanning electron micrograph of red outer-petal epidermis. (**H**) Scanning electron micrograph of yellow inner-petal epidermis.

Subsequently, anatomical structures of petals (Fig. 2A,B) were observed, and the results showed that red pigment was mainly located in the upper and lower epidermis, and sporadic pigment also appeared in the palisade mesophyll and spongy mesophyll, in contrast, the yellow pigment filled up the whole cross section of yellow inner-petal (Fig. 2C,D). Moreover, the epidermal cells of red outer-petal and yellow inner-petal all displayed the colour (Fig. 2E,F). When the epidermal cell shape was examined by scanning electron microscopy, the shapes of red outer-petal was relatively flat while yellow inner-petal displayed a concavoconvex shape, but their interiors showed a similar broken-line arrangement (Fig. 2G,H).

### Cell sap pH

**Figure 3:**
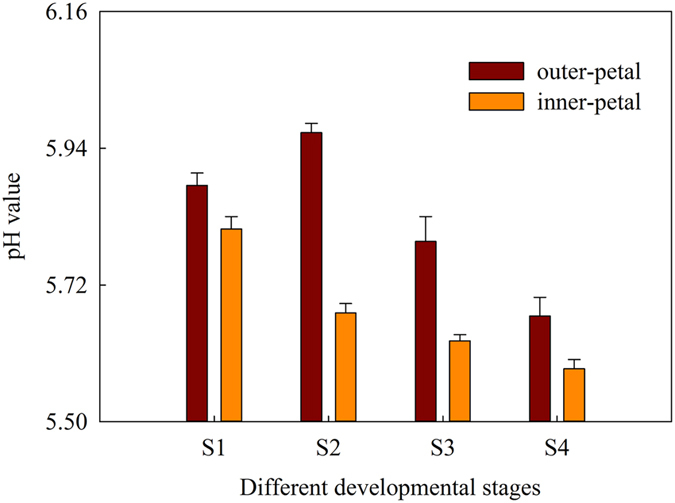
Cell sap pH value of red outer-petal and yellow inner-petal from red outer-petal and yellow inner-petal. S1: Stage 1, flower-bud stage; S2: Stage 2, initiating bloom; S3: Stage 3, bloom stage; S4: Stage 4, wither stage.

The cell sap pH of red outer-petal and yellow inner-petal was showed in Fig. 3, and their range was between 5.59 ± 0.01 and 5.97 ± 0.02, which was slightly acidic. During the developmental stages, the cell sap pH of red outer-petal and yellow inner-petal all exhibited a declining trend of 3.57% and 3.79%, respectively. Meanwhile, the cell sap pH of red outer-petal was always higher than that of yellow inner-petal. Additionally, the correlation coefficient between the cell sap pH value and *H*^*o*^ was only 0.609, and could not reach significant level.

### Organic acids

Considering the contribution of organic acids to cell sap pH, six main organic acids in plants including succinic acid, acetic acid, oxalic acid, malic acid, citric acid and tartaric acid were recorded (Table 1). Among these organic acids, the content of malic acid was lowest, while succinic acid and succinic acid had the relatively higher contents. As a whole, their contents in red outer-petal and yellow inner-petal gradually increased with the flower development, especially acetic acid with an average 194% increase from S1 to S4. For the petal, the contents of succinic acid, acetic acid, oxalic acid, malic acid and tartaric acid in red outer-petal were generally higher than those of yellow inner-petal except at the individual stage, whereas citric acid content displayed the opposite trend. Moreover, the correlation analysis showed that succinic acid, malic acid and tartaric acid were positively correlated with the cell sap pH, but they were not significant.

### Metal elements

Thirteen metal elements were detected in this study using the Inductively Coupled Plasma Emission Spectrometer (ICP) including K, Ca, Mg, Fe, Mn, Zn, Cu, Mo, Al, Na, Ni, Cr and Pb (Table 2). When the content was concerned, K, Ca and Mg had the highest contents in both red outer-petal and yellow inner-petal, followed by Fe, Mn, Zn, Cu, Mo, Al, Na, Ni and Cr, and Pb could not be detected. As far as the petal was concerned, the contents of Ca, Mg, Mn, Zn, Cu and Ni in red outer-petal were always lower than those of yellow inner-petal, whereas only K and Cr showed the opposite trend, Fe, Mo, Al and Na showed no obvious pattern in content level. For the developmental stages, most of metal element contents generally increased in S4 compared with the S1, except for Mo, Ni and Cr. A multiple regression analysis showed that the correlation coefficient between the metal elements and *H*^*o*^ was not significant except for K, Ca, Mg, Mn and Zn.

### Sequence analysis of small RNAs (sRNAs)

**Figure 4:**
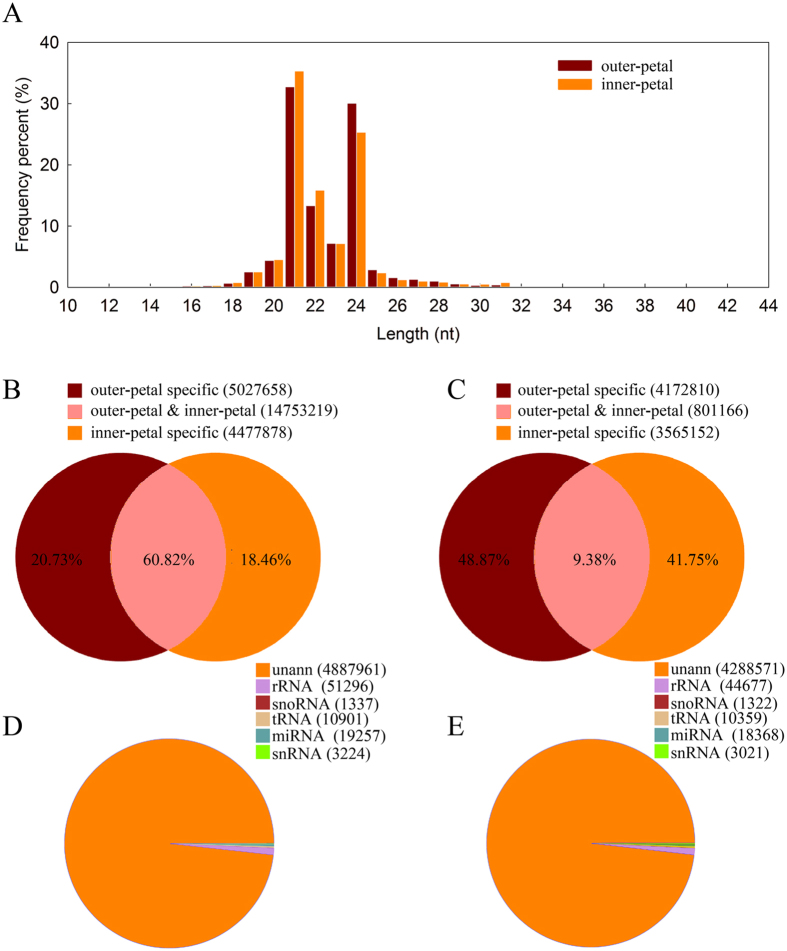
The length distribution of sRNAs (**A**), summary of the common and specific sequences of total sRNAs (**B**) and unique sRNAs (**C**), and mapping statistics of the unique sRNAs between red outer-petal (**D**) and yellow inner-petal (**E**) in *P. lactiflora*.

To understand whether miRNA was involved in the yellow formation of *P. lactiflora*, two independent sRNA libraries from red outer-petal and yellow inner-petal with three biological replicates were generated and sequenced by the high-throughput Illumina sequencing technology. And total of 12,637,298 and 12,353,097 reads were produced from each library, and after removing reads of low-quality, 5′ adapter contaminants, reads without 3′ adapter, reads without inserted fragment, reads containing poly A stretches and reads less than 18 nt, 12,340,077 (97.64%) and 11,918,678 (96.48%) clean reads of 10–44 nt in length were retained in red outer-petal and yellow inner-petal library, respectively (Supplemental Table S1). The overall size distribution pattern of sRNAs was similar between red outer-petal and yellow inner-petal with the majority being 21–24 nt in length (Fig. 4A). The 21-nt sRNAs were the most abundant in both libraries, accounting for 32.74% and 35.33% of the total number of sRNAs of red outer-petal and yellow inner-petal, respectively, followed by 24 nt, 22 nt and 23 nt in that order. Through statistical analysis, the specific unique sRNAs number was 4,172,810 in red outer-petal and 3,565,152 in yellow inner-petal (Fig. 4B), while the specific total sRNAs number was 5,027,658 and 4,477,878, respectively (Fig. 4C). Moreover, these sRNAs were further classified into different RNA categories, 1.34% of red outer-petal and 1.36% of yellow inner-petal unique sRNAs matched to the annotated house-keeping non-coding RNAs, including ribosomal RNA (rRNA), transfer RNA (tRNA), small nuclear RNA (snRNA) and small nucleolar RNA (snoRNA). In addition, 0.39% and 0.42% of the unique sRNAs in red outer-petal and red outer-petal matched with the known miRNAs, respectively (Fig. 4D,E). Similarly, the other two replicates of red outer-petal and yellow inner-petal were showed in Supplemental Table S2, Figure S1 and S2.

### Identification and verification of conserved and novel miRNAs

To identify conserved miRNAs from red outer-petal and yellow inner-petal, sRNA sequences were compared with the known plant miRNAs in the miRBase (Supplemental Material S1). After BLASTn and further sequence analysis, 207 expressed conserved miRNAs were identified in red outer-petal. Moreover, the sequencing frequencies for miRNAs were used as an index for estimating their relative abundance, and the distribution patterns of miRNA frequencies varied greatly. Among them, four miRNAs (miR396b-5p, miR159a, miR5213-5p and miR166a-3p) constituted 66.75% of expressed miRNA reads, and several miRNAs such as miR319, miR1509b, miR8722, miR156e-3p, miR7984a, miR2109 and miR6113 had moderate abundance of expression (12.05%), whereas some miRNAs showed very low expression with several read counts only (miR397, miR8717, miR479, miR8041a-3p and miR5370). Similarly, 204 expressed conserved miRNAs were identified in yellow inner-petal, among them, miR396b-5p, miR159a, miR166a-3p and miR5213-5p had the highest count while miR1446, miR6225-5p and miR482e-5p had the lowest count.

Additionally, 38 and 42 unique sequences were identified as novel miRNAs in red outer-petal and yellow inner-petal, respectively, and their novel miRNA sequences were all 20 to 23 nt in length, with 21 nt being the most abundant in sequencing frequency (Supplemental Material S2). Analysis of these miRNA first nucleotide bias revealed that uridine (U) was the most common nucleotide (Supplemental Figure S3). Moreover, their putative precursors were searched within the corresponding transcriptomes (the accession numbers of ‘Jinhui’ with red outer-petal and yellow inner-petal in NCBI were SRX2473825 and SRX2473826, respectively), and the average length in red outer-petal and yellow inner-petal were all 144 nt, with a range of 58 to 309 nt, and the average minimal free energy (MFE) values were −48.46 and −46.05 kcal/mol, respectively. Likewise, the data of the other two replicates of red outer-petal and yellow inner-petal were showed in Supplemental Material S3, S4 and Figure S3.

In addition, 16 miRNA precursors were further amplified, and the agarose gel electrophoresis confirmed their sizes and expressions, as well as the sequencing validated the identity of their sequences in this study. Of the 16 pre-miRNAs, 14 precursor sequences showed completely consistency with high-throughput sequencing, and 2 precursor sequences were found to have a single nucleotide difference compared with high-throughput sequencing (Supplemental Figure S4).

### Comparative profiling of miRNAs between red outer-petal and yellow inner-petal

In order to identify key miRNAs controlling *P. lactiflora* yellow formation, the comparative profiling of miRNAs between red outer-petal and yellow inner-petal was performed. The comparison of two libraries revealed that 163 differentially expressed conserved miRNAs, of which 83 miRNAs were up-regulated and 80 miRNAs were down-regulated (Supplemental Material S5). In addition, 348 target genes were obtained for 102 differentially expressed miRNAs (Supplemental Material S6). In Gene Ontology (GO) functional annotation, these target genes could be classified into three categories, including biological process [19 GO terms, cellular processes (GO: 0009987) and metabolic processes (GO: 0008152) were dominant functions], cellular component [12 GO terms, cell part (GO: 0044464) and cell (GO: 0005623) were dominant functions] and molecular function [6 GO terms, catalytic activity (GO: 0003824) and binding (GO: 0005488) were dominant functions] (Supplemental Table S3).

Similarly, 28 differentially expressed novel miRNAs were obtained including 10 up-regulated miRNAs and 18 down-regulated miRNAs (Supplemental Material S5). Among them, 72 potential target genes were identified from 25 differentially expressed miRNAs (Supplemental Material S6). GO functional annotation showed that genes involved in metabolic processes (GO: 0008152) and cellular processes (GO: 0009987) were highly represented in biological process, cell (GO: 0005623) and cell part (GO: 0044464) were the most represented GO terms in cellular components, and binding (GO: 0005488) and catalytic activity (GO: 0003824) were the major categories in molecular function category (Supplemental Table S4).

According to the function of target genes, 5 target genes controlling the yellow formation were screened including squamosa promoter binding protein-like gene (*SPL*), *SPL1*^31^, v-myb myeloblastosis viral oncogene homolog (avian) 114 gene (*MYB114*)^32^,^33^, flavonoid 3-*O*-glucosyltransferase gene (*F3GT*) and *F3GT7*^11^,^34^, and the corresponding miRNAs were miR156d, miR156e-3p, miR828a, miR2616 and novel-miR25, respectively. And we found that each miRNA had one or more target genes, but some genes were not related with the colour formation, and the details were all listed in Supplemental Table S5. When the red outer-petal was the control group, the expression levels of candidated miRNAs were all down-regulated in yellow inner-petal, and their corresponding target genes *SPL, SPL1, MYB114* and *F3GT7* were all up-regulated while *F3GT* was down-regulated.

### Expression pattern of miRNAs and their target genes by Q-PCR

**Figure 5:**
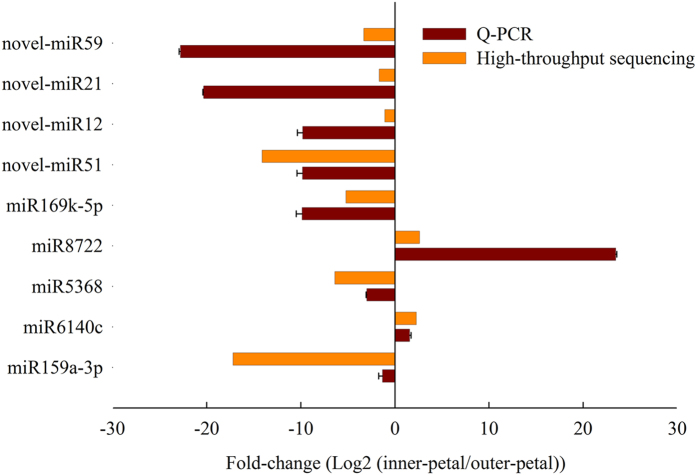
Verification of the miRNAs expression between red outer-petal and yellow inner-petal by Q-PCR. The x-axis shows the Log2 ratio of their expression in outer-petal versus inner-petal, and the y-axis shows the miRNAs validated in this study.

To validate the reliability of the sequencing results, based on the fourteen pre-miRNA sequences, 9 miRNAs (5 known and 4 new miRNAs) were selected to analyse their expression patterns by Q-PCR. As shown in Fig. 5, the expression levels of miR6140c and miR5368 were up-regulated while miR159a-3p, miR5368, miR169a-5p, novel-miR59, novel-miR12, novel-miR21 and novel-miR51 were down-regulated in yellow inner-petal compared with red outer-petal, which was consistent with the results from high-throughput sequencing.

**Figure 6:**
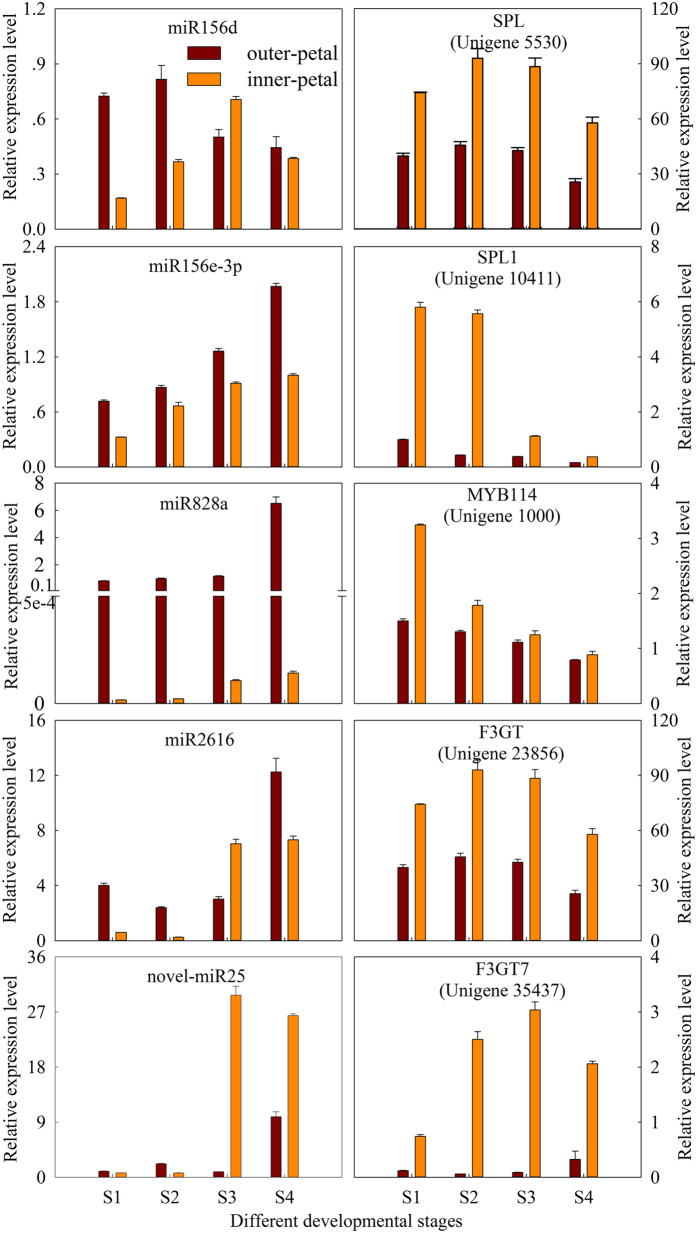
Dynamic expression patterns of candidate miRNAs and their corresponding target genes during the flower development. S1: Stage 1, flower-bud stage; S2: Stage 2, initiating bloom; S3: Stage 3, bloom stage; S4: Stage 4, wither stage.

To understand the role of miRNAs in *P. lactiflora* yellow formation, the dynamic expression patterns of the above 4 miRNAs and their corresponding target genes during the flower development were detected by stem-loop Q-PCR. As shown in Fig. 6, the expression levels of miR156d in red outer-petal and yellow inner-petal all increased at first and then had a descending tendency. Meanwhile, miR156e-3p expression kept increasing with the flower development, and it was highly expressed in red outer-petal. And their corresponding target genes displayed a different expression pattern. *SPL1* expression was markedly down, and its higher expression was in yellow inner-petal. *SPL* expression had a decreasing trend generally, and the latter was more obvious. miR828a expression level increased with the flower development, and the expression level in S4 was more than 8.0 times higher than that in S1. However, the expression level of miR828a in red outer-petal was an average of 37,236.8 times higher than that in yellow inner-petal. miR828a corresponding target gene *MYB114* showed the decreased expression during the developmental stages, and its level in yellow inner-petal was always higher than that in red outer-petal. The expression level of miR2616 increased with the flower development, and was characterized by an initial decrease followed by an increasing trend, and the expression tendency of its target gene *F3GT* expression was first rise and then gradually decrease. In addition, novel-miR25 and its target gene *F3GT7* basically showed up-regulated expression patterns, and only the expression levels of red outer-petal and yellow inner-petal in S1 and S2 had the opposite trend. These results exhibited the negative relationship between the expression patterns of miR156e-3p, miR828a and miR2616 and their corresponding target genes *SPL1, MYB114* and *F3GT*; moreover, *SPL1* and *F3GT7* exhibited no significant relationship to the expression of miR156e-3p and novel-miR25, which strongly suggested that the miR156e-3p, miR828a and miR2616 might be involved in the yellow formation of *P. lactiflora* inner-petal.

## Discussion

Flower colour formation is very complicated and involves many different mechanisms^3^. Among them, the distribution and type of pigments are crucial^35^. Our previous study indicated that the flavonoid compositions of *P. lactiflora* outer-petal and inner-petal were identical, and their contents were basically higher in outer-petal, while the co-pigmentation effect of anthoxanthins caused the inner-petal to be yellow^11^. Besides the pigment, petal tissue structure, cell sap pH and metal elements are all required for the expression of petal colour^35^. Qi *et al*.^36^ found that the blue cells of the pale blue flowers in grape hyacinth (*Muscari latifolium* Kirk and M. bourgaei Baker) are located only in the palisade mesophyll, while the cells in the purple flowers are mainly in the lower epidermis and the sub-epidermal layer which contributed to the much deeper colour of the purple flowers. Noda *et al*.^37^ found that the cell shape influenced the optical properties in *Antirrhinum majus*, the magenta epidermal cells were conical and the pink epidermal cells were flat. Avila-Rostant *et al*.^38^ speculated that there was correspondence between the lightness of the pigmentation and the vacuolar pH values with the lighter colours having higher pH values in anthurium (*Anthurium andraeanum* Hort.). In addition, ferric ion was correlated with the blue in blue poppy (*Meconopsis grandis* Prain)^39^. In the present study, *H°* indicated that the colours of the outer-petal and inner-petal were reddish-purple and yellow in S1, and then faded gradually as flower development proceeded, which was in agreement with our previous results^11^. And the anatomical structures of petals showed that epidermal cells of red outer-petal and yellow inner-petal were filled with the pigments, which was also confirmed by the observation of the cross section. Compared with the red outer-petal, the yellow pigment filled up the whole cross section of yellow inner-petal, and its epidermal cell shape was concavoconvex, which might contribute to the much deeper colour of yellow inner-petal. Moreover, a significant correlation could not be observed between the pH value and *H*^*o*^ suggesting that the colour in *P. lactiflora* was not heavily influenced by the cell sap pH, and it had little relationship with our measured organic acids. Furthermore, 13 metal elements detected in red outer-petal and yellow inner-petal did not represent significant correlation with *H*^*o*^ except for K, Ca, Mg, Mn and Zn, and the roles of these metal elements in the yellow colour formation of *P. lactiflora* remained to be explored further.

Identification of numberous miRNAs using miRNA-Seq has attracted much attention during the past few years, and more and more studies on miRNAs involved in plant development and morphogenesis processes have been reported^15^. As an important ornamental flower, *P. lactiflora* has been widely used for urban greening and high-grade cut flower, and understanding the functions of miRNAs in regulating the formation of its rare yellow colour is of great value. In the present study, under the consistent genetic background, total of 12,340,077 and 11,918,678 clean reads were generated, subsequently, 4,172,810 and 3,565,152 specific unique sRNAs were obtained from the red outer-petal and yellow inner-petal, respectively. The number of sRNAs was far less than the report of miRNAs responsive to *Botrytis cinerea* in *P. lactiflora*^21^, which demonstrated that the mechanism of colour formation was less complicated compared with the stress response in *P. lactiflora*. With respect to the length distribution of sRNAs, 21–24 nt sRNAs dominated the sRNA transcriptome in red outer-petal and yellow inner-petal with the 21-nt sRNAs being the most abundant in length, which was in accordance with the reports in *P. lactiflora*^21^, *P. persica*^17^ and *C. indica*^22^, while not in agreement with 24-nt sRNAs being the most abundant in *C. tinctorius*^16^ and *D. morifolium*^20^. This was mainly because of the plant species and the specific enzymes that were used to process the different length of sRNAs^40^. Moreover, the conserved and novel miRNAs were identified. And in many studies, a large number of miRNAs were identified from plant species with known genome sequence, such as arabidopsis (*Arabidopsis thaliana* (L.) Heynh)^41^, mei (*Prunus mume* Sieb. et Zucc.)^42^, rice (*Oryza sativa* L.)^43^, etc. Here, 207 and 204 expressed conserved miRNAs were identified in red outer-petal and yellow inner-petal by comparing with the known plant miRNAs in the miRBase due to *P. lactiflora* without genome sequences. Meanwhile, 38 and 42 novel miRNAs were identified in red outer-petal and yellow inner-petal, respectively. Due to lack of *P. lactiflora* genome sequences, miRNAs precursors were predicted based on corresponding ‘Jinhui’ transcriptomes, and this method was also used in *P. lactiflora* previously with good results^21^. On this basis, subcloning and sequencing further validated the precursor sequences of 14 miRNAs, and the other 2 precursor sequences had a single nucleotide difference compared with high-throughput sequencing, which indicated that they were authentic miRNAs, and the high-throughput sequencing could accurately identify miRNAs from *P. lactiflora*.

Analysis of differentially expressed miRNAs in two contrasting flower colour materials could provide further insight into the colour formation from the post-translational level. In this study, a total of 163 differentially expressed conserved miRNAs and 28 differentially expressed novel miRNAs were obtained when the red outer-petal served as the control. Moreover, 348 and 72 target genes were obtained for 102 differentially expressed conserved miRNAs and 25 differentially expressed novel miRNAs, respectively. According to the function of target genes, 5 target genes related to the colour formation were screened, and the 5 corresponding miRNAs were obtained. And the dynamic expression levels of these 5 target genes in yellow inner-petal were always higher than those in red outer-petal during the flower development. In previous studies, Gou *et al*.^31^ found that *SPL9* negatively regulated anthocyanin accumulation in *A. thaliana* by directly preventing expression of anthocyanin biosynthetic genes, especially *DFR*, and *SPL* was targeted by miR156. In *P. lactiflora*, miR156e-3p expression kept increasing with the flower development, and it was highly expressed in red outer-petal; the corresponding target gene *SPL1* (Unigene 10411) expression was markedly reduced, and its higher expression was in yellow inner-petal; and the total contents of anthocyanins in red outer-petal were all higher compared to the yellow inner-petal^11^, which all indicated that the yellow formation might be under the regulation of miR156e-3p-targeted *SPL1* (Unigene 10411). And our previous study showed that *PlPAL, PlFLS, PlDFR, PlANS, Pl3GT* and *Pl5GT* were highly expressed in red outer-petal, which inducing inhibition of anthocyanin biosynthesis mediated yellow formation in *P. lactiflora*^11^. These suggested that *SPL1* mgiht negatively regulated anthocyanin accumulation by directly preventing expression of anthocyanin biosynthetic genes, but which gene could need further analysis. Moreover, previous studies all suggested that *MYB114* positively regulated anthocyanin accumulation in *A. thaliana*^32^,^33^. Here, the negative correlation was found between the expression levels of *MYB114* and of its corresponding miR828a, but the expression levels of miR828a were always higher in red outer-petal, which was opposite with the report in yellow and red *C. indica*^22^. Additionally, Panumas *et al*.^34^ found that the lack of *F3GT* expression induced the lack of anthocyanins in Malay apple (*Syzygium malaccense* (L.) Merr. & L. M. Perry), and we also found that *F3GT* had higher expression levels in red outer-petal compared to the inner-petal in during the developmental stages^11^. However, the identified *F3GT* and *F3GT7* were all heavily expressed in yellow inner-petal, which might be because *F3GT* was a large family, these *F3GT* and *F3GT7* might not involve in the flavonoid biosynthesis pathway. Nevertheless, further analysis of the differentially expressed miR156e-3p and its corresponding target gene *SPL1* are still necessary to explore the miRNA-mediated regulatory mechanisms of the yellow formation in *P. lactiflora*.

In conclusion, the yellow formation was influenced by the anatomical structures of the petal, and not by the cell sap pH and metal elements. Moreover, miRNA-Seq and the comparative profiling of miRNAs provided an extensive perspective into the yellow formation in *P. lactiflora*. Many conserved and novel miRNAs were identified, 5 differentially expressed miRNAs and their corresponding target genes related to yellow formation were screened, and further investigation confirmed the yellow formation might be under the regulation of miR156e-3p-targeted *SPL1* (Unigene 10411). These results provided valuable information for understanding the miRNA-mediated regulating mechanism of the yellow formation in *P. lactiflora*.

## Materials and Methods

### Plant materials

*P. lactiflora* was grown in the germplasm repository of Horticulture and Plant Protection College, Yangzhou University, Jiangsu Province, China (32°30′ N, 119°25′ E). A bicoloured flowers cultivar ‘Jinhui’ with red outer-petal and yellow inner-petal was used as the experimental material, and the ground plants grew well with sufficient light and water supply. The samples were taken from March to May 2014, which could be divided into four developmental stages including flower-bud stage (S1), initiating bloom stage (S2), bloom stage (S3) and wither stage (S4). Among these samples, the young outer-petal and inner-petal samples with significantly different colours were used for small RNA sequencing, and the developmental petals were used for physiological indices measurement and expression analysis of candidated genes. After the colour indices measurement and anatomy observations, all samples were immediately frozen in liquid nitrogen and stored at −80 °C until further analysis.

### Colour indices measurement

The colour indices of fresh petals were measured with a hand-held RM200QC spectrocolourimeter (X-Rite, Switzerland) using two colour parameters including a* and b* values. The hue angle (*H°* = arctangent (b*/a*)) were calculated.

### Anatomy observations

Microscopic observation of outer epidermal cells and transverse sections were performed using free-hand sections. The petals were firstly cross-sectioned, and an epidermal layer was subsequently peeled off which were placed on a glass slide with a drop of water. Moreover, they were immediately observed by a light microscope (Olympus CX31RTSF, Tokyo, Japan). Moreover, the shape of epidermal cells were observed by the environmental scanning electron microscopy (Philips XL-30 ESEM, Amsterdam, The Netherlands), and the specific operational method was referred to the report of Zhao *et al*.^44^.

### Cell sap pH determination

Cell sap pH of petals was determined according to the report of Pei *et al*.^45^ with some modifications. 3.0 g petals of each sample were ground with liquid nitrogen and centrifuged at 12,000 rpm for 20 min. The supernatant was transferred to 10 mL centrifuge tube, and then a Sartorius PB-10 pH meter (Beijing Sartorius Scientific Instrument Corporation) was used to determinate its pH value.

### Metal elements measurement

The petals of each sample were firstly put in oven for 10 min at 105 °C after washing with deionized water, and then dried to constant weight at 70 °C as well as ground into powder. Subsequently, 5 mL HNO_3_, 3 mL ultrapure water and 2 drops of H_2_O_2_ were added to 0.5 g powder, which were decomposed with the microwave digestion instrument (MARS6, CEM, USA). After digestion, the solution was diluted with ultrapure water to 50 mL. On this basis, 9 mL ultrapure water was added to 1 mL analyzed solution, and its metal elements were measured using ICP (iCAP6300, Thermo Fisher, USA).

### Organic acids measurement

3.0 g petals of each sample were ground with liquid nitrogen and centrifuged at 12,000 rpm for 20 min, and the supernatant was transferred to 1.5 mL centrifuge tube. Based on this, 200 μL supernatant and 800 μL ultrapure water were transferred to 2 mL centrifuge tube together, which was passed through 0.22 μm membrane filters (Shanghai ANPEL Scientific Instrument Co., Ltd., China). This experiment was performed three times for each sample, and the extracts were used for analysis of organic acids. Qualitative and quantitative analysis of organic acids was performed using Ion Chromatograph System (ICS-2100, Thermo, USA). The anion exchange column was Ion Pac AS11-HC (2.0 mm × 50 mm) and AG11-HC (2.0 mm × 250 mm) (Dionex, USA). The column temperature was 30 °C, injected volume was 25 μL, flow rate was 0.3 mL/min and the electric current of suppressor was 50 mA. In addition, the eluent was KOH with 1.0 mmol/L at 0 min, 1.0 mmol/L at 8 min, 30.0 mmol/L at 28 min, 50.0 mmol/L at 35 min, 50.0 mmol/L at 40 min, 1.0 mmol/L at 41 min, and then returned to 1.0 mmol/L at 55 min.

### Small RNA library construction and sequencing

Total RNA was extracted according to a modified CTAB extraction protocol^46^. Prior to small RNA library construction, RNA samples were examined by a spectrophotometer (Eppendorf, Germany) and 1% agarose gel electrophoresis. Moreover, RNA fragments of 18–30 nt long were separated from total RNA using polyacrylamide gel electrophoresis. The Solexa adaptors were added to the fragments at both 5′- and 3′-ends, and they were converted to cDNA according to reverse transcription PCR kit (Invitrogen, USA). The purified fragments were sent to sequence at Beijing Genomic Institute (Shenzhen, China) using an Illumina HiSeq™ 2000 platform (Illumina Inc., San Diego, CA, USA). The data of ‘Jinhui’ with red outer-petal and yellow inner-petal were submitted to the National Center for Biotechnology Information (NCBI) under accession numbers SRX1996909 and SRX1996992, respectively.

### miRNAs identification and their target genes prediction

After getting rid of impure sequences (low quality reads, reads with 5′ primer contaminants, reads without 3′ primer, reads without the insert tag, reads with poly A and reads shorter than 18 nt), unique reads were screened against GenBank including rRNA and tRNA, as well as Rfam (http://rfam.sanger.ac.uk) to remove non-coding RNA, such as rRNA, tRNA, snRNA and snoRNA. After these, the merged unique reads were also screened against the miRNA database (miRBase 21.0) using a nucleotide-nucleotide Basic Local Alignment Search Tool (BLASTn) to identify the conserved miRNAs^47^. Meanwhile, in order to identify the novel miRNAs, all candidated precursors with hairpin-like structures were obtained using the Mireap program (http://sourceforge.net/projects/mireap). Additionally, the unigene sequences of the corresponding ‘Jinhui’ transcriptomes were used to predict the target genes of miRNAs by the psRNA Target program (http://plantgrn.noble.org/psRNATarget) with the default parameters. The specific methods were referred to Allen *et al*.^48^ and Schwab *et al*.^49^.

### Differentially expressed miRNAs and their target genes annotation

miRNAs expression levels were calculated according the value of Reads Per Million reads (RPM). Differentially expressed miRNAs were defined based on P value ≤ 0.05 and differential expression fold >2. Functional annotation of target genes was performed using various bioinformatics procedures, including GO.

### Subcloning of pre-miRNA sequences

Total RNA was extracted as described above, and the cDNA was synthesized from 1 μg RNA using PrimeScript^®^ RT reagent Kit With gDNA Eraser (TaKaRa, Japan). Sixteen pairs of primers for obtaining pre-miRNA sequences were listed in Supplemental Table S6. Polymerase chain reaction (PCR) contained 2.5 μL cDNA, 2 μL mix solution of target gene primers, 2.5 μL 10× TransTaq^®^ HiFi Buffer II, 2 μL dNTPs (2.5 mM), 0.3 μL TransTaq^®^ HiFi DNA Polymerase (TransGen, China), 15.7 μL of ddH_2_O. And the amplification was performed under the following conditions: 94 °C denaturation for 3 min, 35 running cycles of 94 °C for 30 s, 55.2 °C for 30 s, 72 °C for 30 s, and an elongation cycle of 72 °C for 10 min. PCR products were separated by 3% agarose gel electrophoresis, and the incised gels were purified using a TaKaRa MiniBEST Agarose Gel DNA Extraction Kit Ver.3.0 (TaKaRa, Japan). The extracted products were cloned into p*EASY*^TM^-T5 Zero vector (TransGen, China) and transformed into competent *Escherichia coli Trans*1-T1 cells (TransGen, China). The recombinant plasmids were sent Shanghai Sangon Biological Engineering Technology & Services Co., Ltd. (Shanghai, China) to sequence.

### Gene expression analysis

Gene transcript levels were analysed using Q-PCR with a BIO-RAD CFX Connect^TM^ Optics Module (Bio-Rad, USA). miRNA was extracted according to RNAiso for Small RNA (TaKaRa, Japan), and two methods were used to detect their expression patterns. Based on the obtained fourteen pre-miRNA sequences, nine miRNAs were randomly selected to verify their expression. The reverse-transcription of miRNA was performed by PrimeScript^®^ RT reagent Kit With gDNA Eraser (TaKaRa, Japan), and the specific primers were the same as subcloning primers (Supplemental Table S6). Meanwhile, the screened miRNAs according to the function of target genes from differentially expressed miRNAs were expressed, and its reverse-transcription was performed by miRNA First Strand cDNA Synthesis Kit (Sangon, China), and the specific primers were listed in Supplemental Table S7. On this basis, Q-PCR was performed using TransStart Tip Green qPCR SuperMix (TransGen, China) and contained 2× TransStart Tip Green qPCR SuperMIx 12.5 μL, 2 μL cDNA solution as a template, 2 μL mix solution of target gene primers and 8.5 μL ddH_2_O in a final volume of 25 μL. The amplification was carried out under the following conditions: 95 °C for 30 s followed by 40 cycles at 95 °C for 5 s, 55.2 °C for 30 s and 72 °C for 30 s. *P. lactiflora Actin* (JN105299) was used as an internal control, and all gene-specific primers for Q-PCR were shown in Supplemental Table S8. Gene relative expression levels of target genes were calculated by the 2^−△△Ct^ comparative threshold cycle (Ct) method^50^.

## Additional Information

**How to cite this article:** Zhao, D. *et al*. Identification and comparative profiling of miRNAs in herbaceous peony (*Paeonia lactiflora* Pall.) with red/yellow bicoloured flowers. *Sci. Rep.* **7**, 44926; doi: 10.1038/srep44926 (2017).

**Publisher's note:** Springer Nature remains neutral with regard to jurisdictional claims in published maps and institutional affiliations.

## Supplementary Material

Supplementary 1

Supplementary Material S1

Supplementary Material S2

Supplementary Material S3

Supplementary Material S4

Supplementary Material S5

Supplementary Material S6

## Figures and Tables

**Table 1:** Contents of organic acids in red outer-petal and yellow inner-petal during the flower development (μg g^−1^ FW).

organic acids	S1		S2		S3		S4	
	outer-petal	inner-petal	outer-petal	inner-petal	outer-petal	inner-petal	outer-petal	inner-petal
succinic acid	33.49 ± 4.24	10.58 ± 0.06	32.51 ± 4.15	9.22 ± 0.22	38.89 ± 5.03	12.93 ± 0.04	62.40 ± 2.04	25.64 ± 0.20
acetic acid	6.66 ± 3.71	8.12 ± 0.20	15.27 ± 6.22	6.62 ± 1.09	18.13 ± 7.08	11.26 ± 0.20	22.46 ± 0.65	20.43 ± 0.34
oxalic acid	24.17 ± 1.59	22.38 ± 0.63	28.99 ± 5.02	18.94 ± 0.84	31.47 ± 0.21	26.02 ± 0.02	35.35 ± 1.14	37.88 ± 0.27
malic acid	2.26 ± 0.54	—	2.36 ± 0.39	—	2.56 ± 0.49	—	3.90 ± 0.64	—
citric acid	10.24 ± 1.30	15.20 ± 2.66	14.94 ± 3.73	12.31 ± 0.89	18.73 ± 1.03	19.34 ± 0.52	23.26 ± 3.30	24.98 ± 1.51
tartaric acid	16.25 ± 1.55	10.15 ± 0.04	20.38 ± 1.82	7.66 ± 0.75	24.05 ± 3.02	12.74 ± 0.03	21.71 ± 2.18	23.54 ± 1.75

S1: Stage 1, flower-bud stage; S2: Stage 2, initiating bloom; S3: Stage 3, bloom stage; S4: Stage 4, wither stage. —: not detected.

**Table 2:** Contents of metal elements in red outer-petal and yellow inner-petal during the flower development (μg g^−1^ DW).

metal elements	S1		S2		S3		S4	
	outer-petal	inner-petal	outer-petal	inner-petal	outer-petal	inner-petal	outer-petal	inner-petal
K	19,935.00 ± 95.00	11,550.00 ± 10.00	18,960.00 ± 20.00	11,450.00 ± 50.00	20,465.00 ± 125.00	12,105.00 ± 115.00	20,135.00 ± 75.00	13,870.00 ± 110.00
Ca	2,306.00 ± 5.00	5,225.50 ± 94.50	2,082.00 ± 29.00	5,692.50 ± 1.50	2,267.00 ± 10.00	5,571.50 ± 72.50	3,312.50 ± 20.50	8,004.50 ± 47.50
Mg	1,651.50 ± 12.50	2,441.50 ± 10.50	1,562.50 ± 7.50	2,466.50 ± 12.50	1,673.00 ± 6.00	2,466.00 ± 4.00	1,740.50 ± 7.50	2,859.00 ± 15.00
Fe	55.90 ± 3.60	51.00 ± 0.50	59.85 ± 0.05	108.20 ± 1.40	65.10 ± 1.80	53.35 ± 0.65	150.90 ± 1.90	89.65 ± 2.05
Mn	7.65 ± 0.05	13.90 ± 0.20	7.85 ± 0.25	14.90 ± 0.20	8.10 ± 0.20	13.10 ± 0.00	10.85 ± 0.25	18.00 ± 0.40
Zn	35.45 ± 0.15	43.70 ± 0.10	35.65 ± 0.05	45.30 ± 1.30	39.90 ± 1.50	42.90 ± 0.30	40.70 ± 0.60	56.80 ± 0.10
Cu	9.85 ± 0.05	12.60 ± 0.60	10.60 ± 0.70	13.50 ± 0.10	11.25 ± 1.25	11.90 ± 0.40	12.95 ± 0.15	14.85 ± 0.05
Mo	0.45 ± 0.05	0.30 ± 0.10	0.55 ± 0.05	1.15 ± 0.15	0.35 ± 0.05	0.35 ± 0.05	0.25 ± 0.05	0.20 ± 0.00
Al	23.55 ± 0.65	30.10 ± 10.30	38.75 ± 0.75	23.85 ± 3.05	34.65 ± 0.35	21.30 ± 3.00	123.70 ± 0.10	59.55 ± 1.45
Na	21.15 ± 0.00	12.75 ± 0.00	26.25 ± 0.00	105.60 ± 0.00	36.45 ± 0.00	73.80 ± 0.00	38.25 ± 0.00	60.85 ± 0.00
Ni	1.75 ± 0.05	2.50 ± 0.10	3.55 ± 0.05	11.85 ± 0.25	2.45 ± 0.35	1.85 ± 0.15	—	—
Cr	6.10 ± 0.10	5.85 ± 0.45	5.65 ± 0.45	5.65 ± 0.45	5.45 ± 0.35	4.85 ± 1.05	5.50 ± 0.50	4.45 ± 0.15
Pb	—	—	—	—	—	—	—	—

S1: Stage 1, flower-bud stage; S2: Stage 2, initiating bloom; S3: Stage 3, bloom stage; S4: Stage 4, wither stage. — not detected.
