# Supplementary material for: Identification and comparative profiling of miRNAs in herbaceous peony (Paeonia lactiflora Pall.) with red/yellow bicoloured flowers
Source: Sci Rep. 2017 Mar 20;7:44926. doi: 10.1038/srep44926 (PMC5357961; doi:10.1038/srep44926)
Supplement: Supplementary 1 [file srep44926-s1.doc]

Identification and comparative profiling of miRNAs in herbaceous peony (*Paeonia lactiflora* Pall.) with red/yellow bicoloured flowers

Daqiu Zhao, Mengran Wei, Min Shi, Zhaojun Hao, Jun Tao*


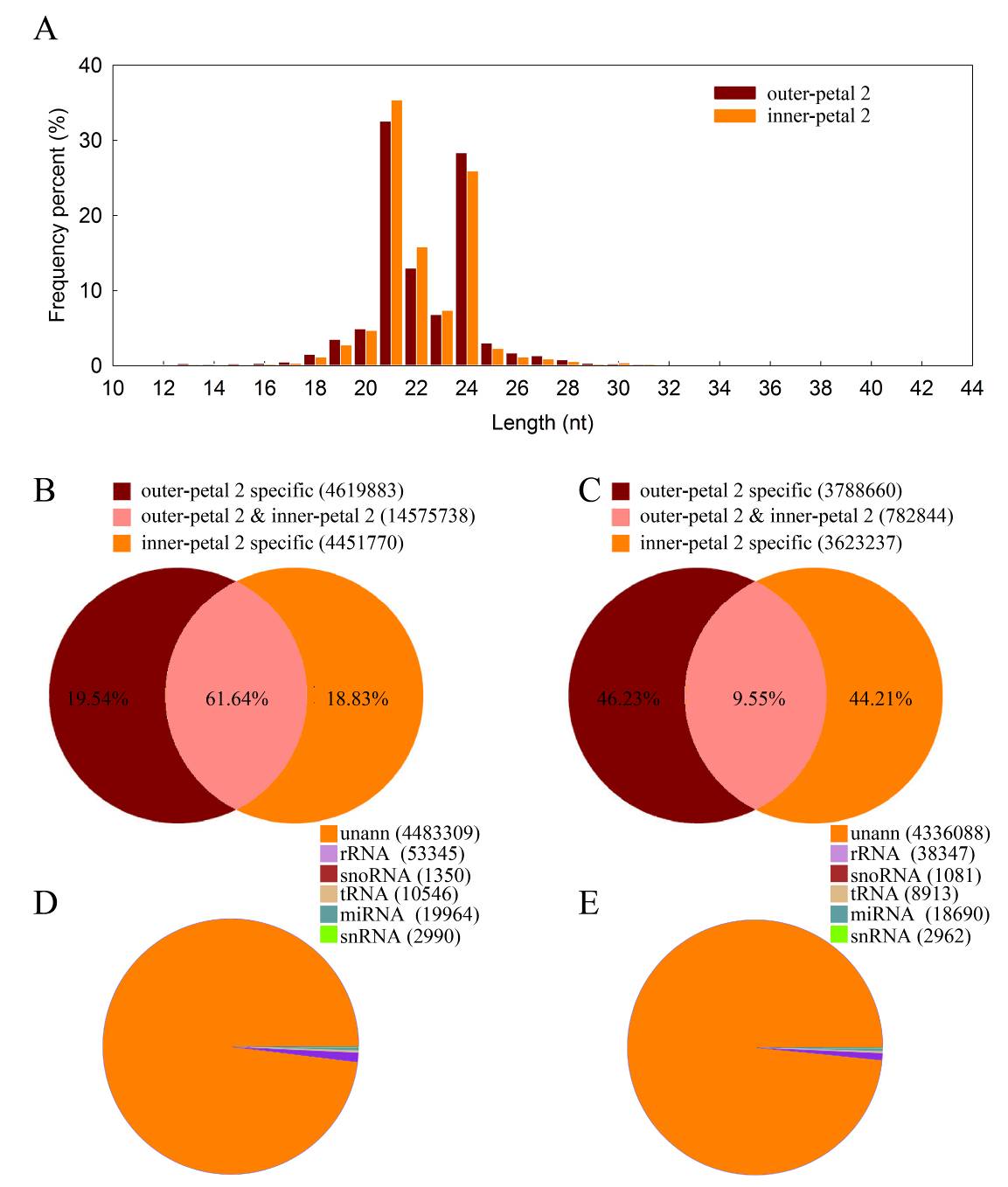


**Supplemental Figure S1. The length distribution of sRNAs (A), summary of the common and specific sequences of total sRNAs (B) and unique sRNAs (C), and mapping statistics of the unique sRNAs between red outer-petal 2 (D) and yellow inner-petal 2 (E).**


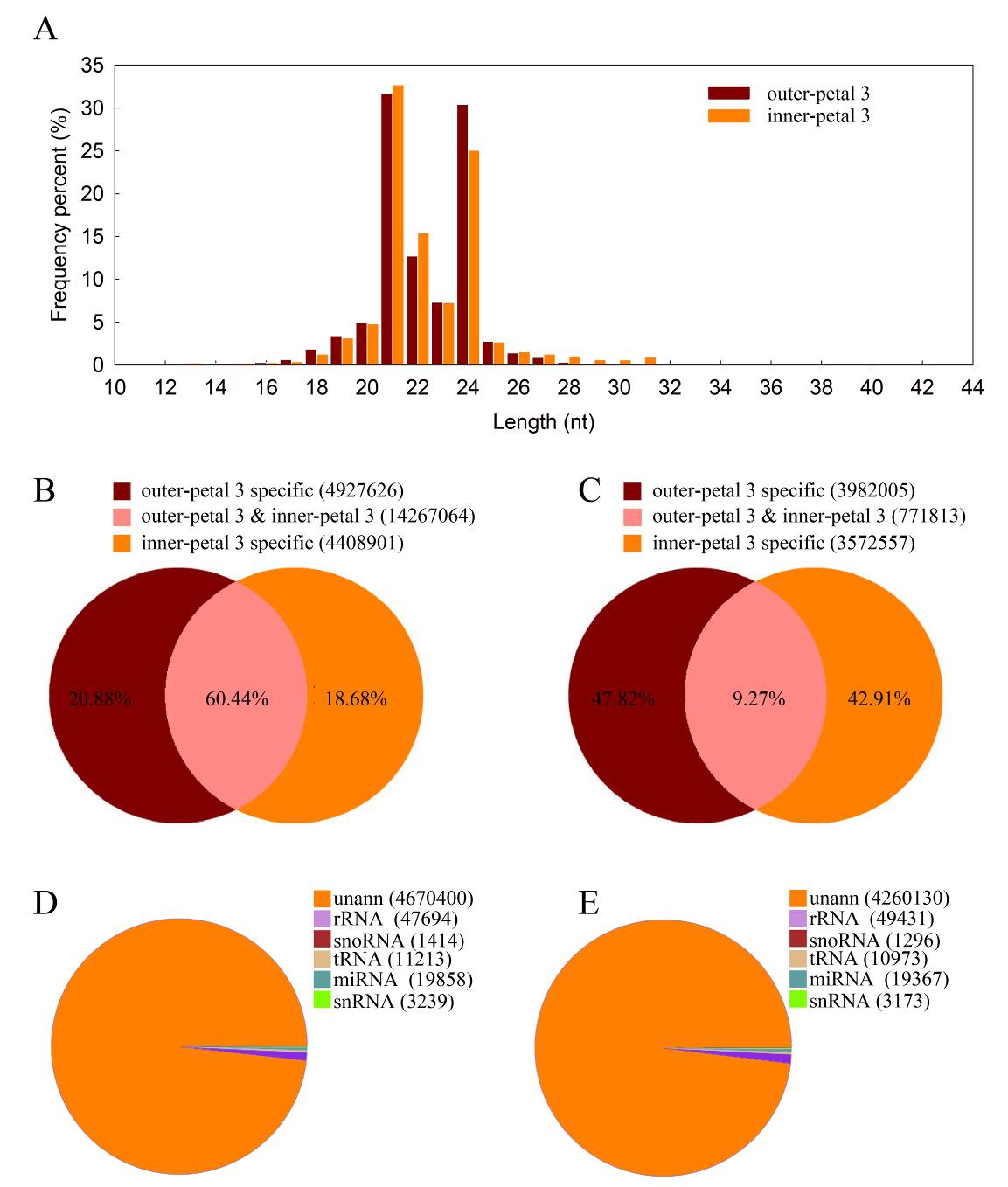


**Supplemental Figure S2. The length distribution of sRNAs (A), summary of the common and specific sequences of total sRNAs (B) and unique sRNAs (C), and mapping statistics of the unique sRNAs between red outer-petal 3 (D) and yellow inner-petal 3 (E).**

outer-petal 1 and inner-petal 1


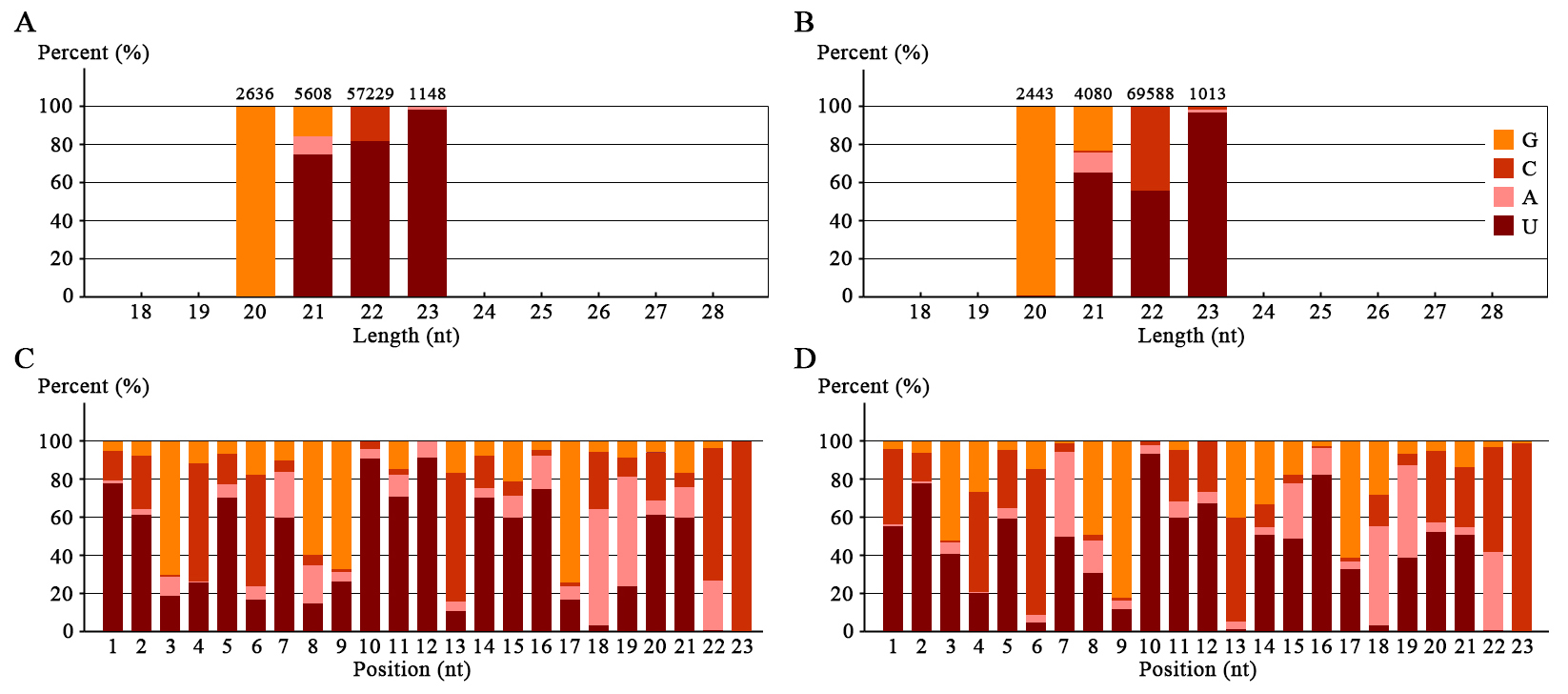


outer-petal 2 and inner-petal 2


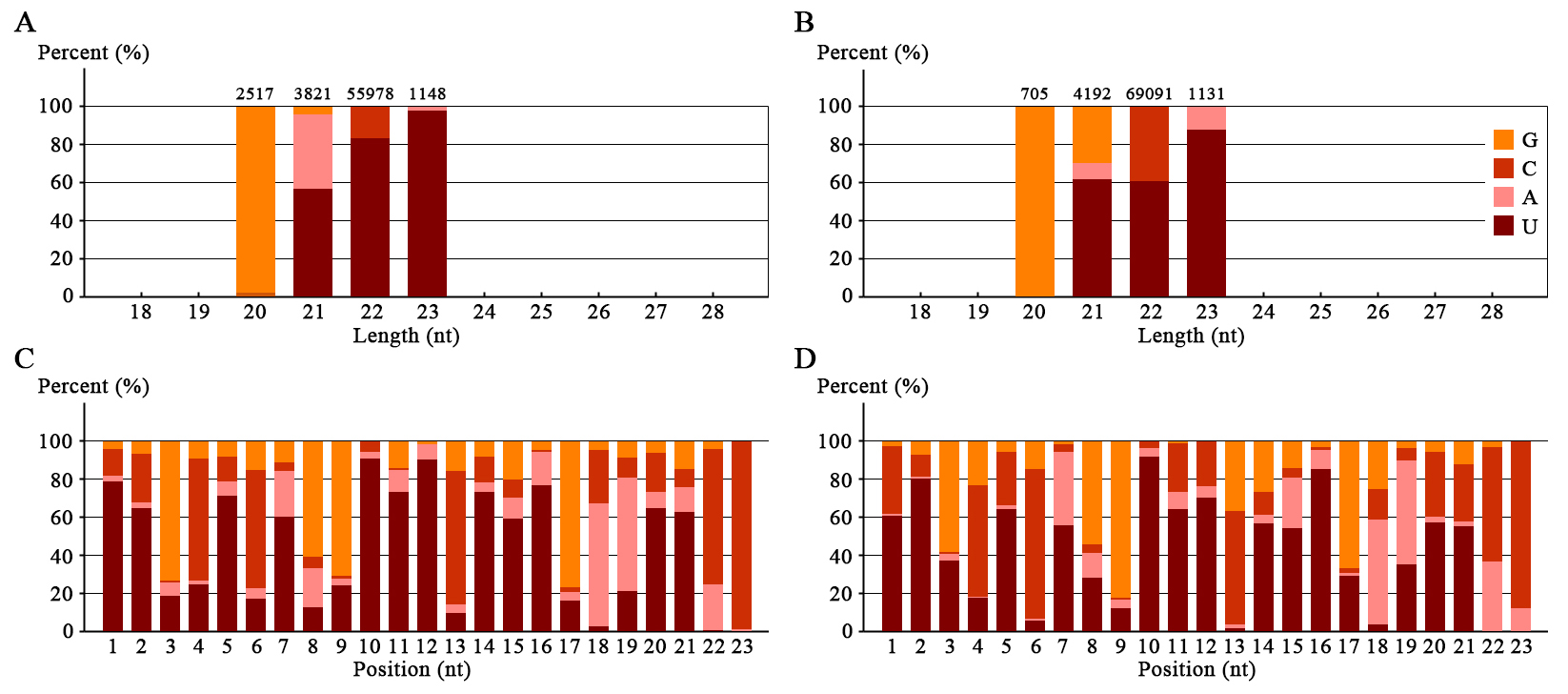


outer-petal 3 and inner-petal 3


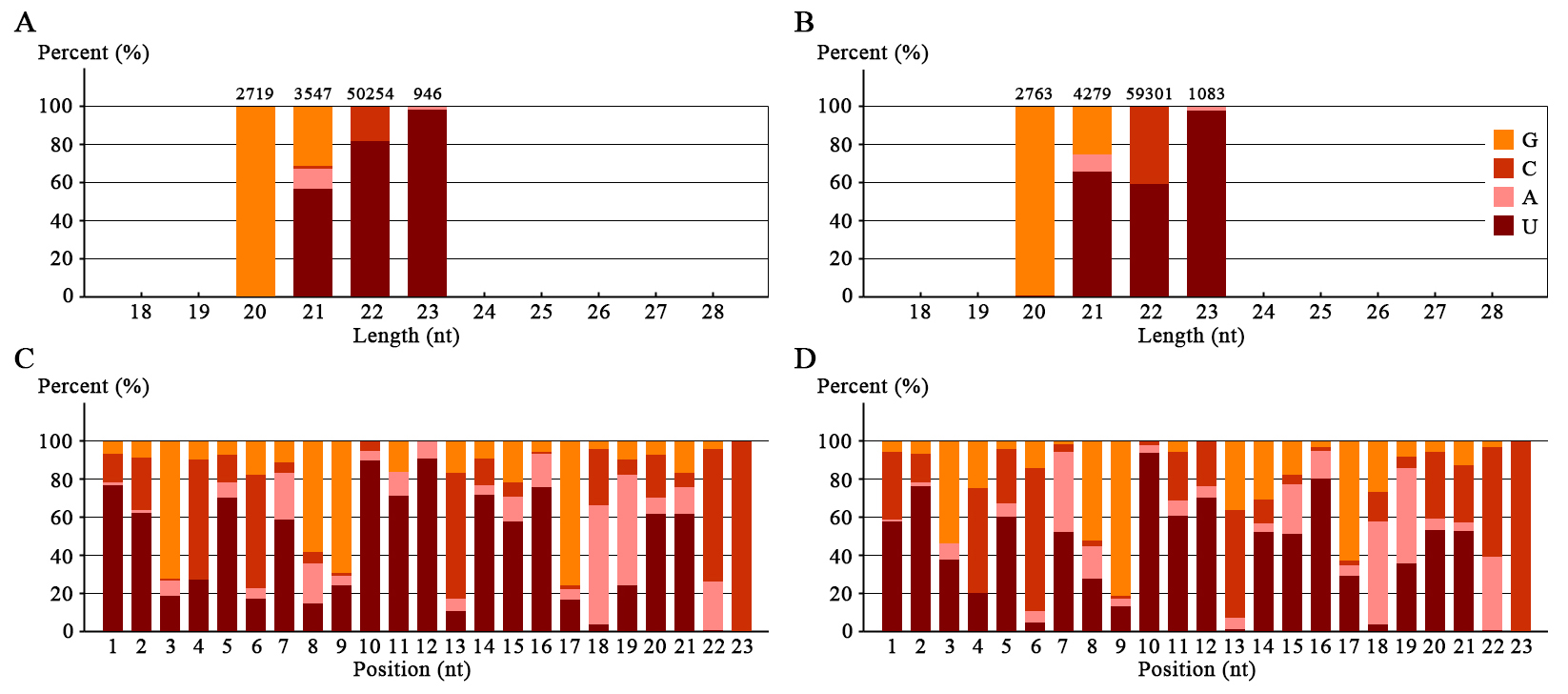


**Supplemental Figure S3. Analysis of miRNAs nucleotide bias in two libraries from red outer-petal and** **yellow inner-petal.** (**A**) Analysis of first nucleotide bias of miRNAs in red outer-petal. (**B**) Analysis of first nucleotide bias of miRNAs in yellow inner-petal. (**C**) Analysis of each nucleotide bias of miRNAs in red outer-petal. (**D**) Analysis of each nucleotide bias of miRNAs in yellow inner-petal.

A


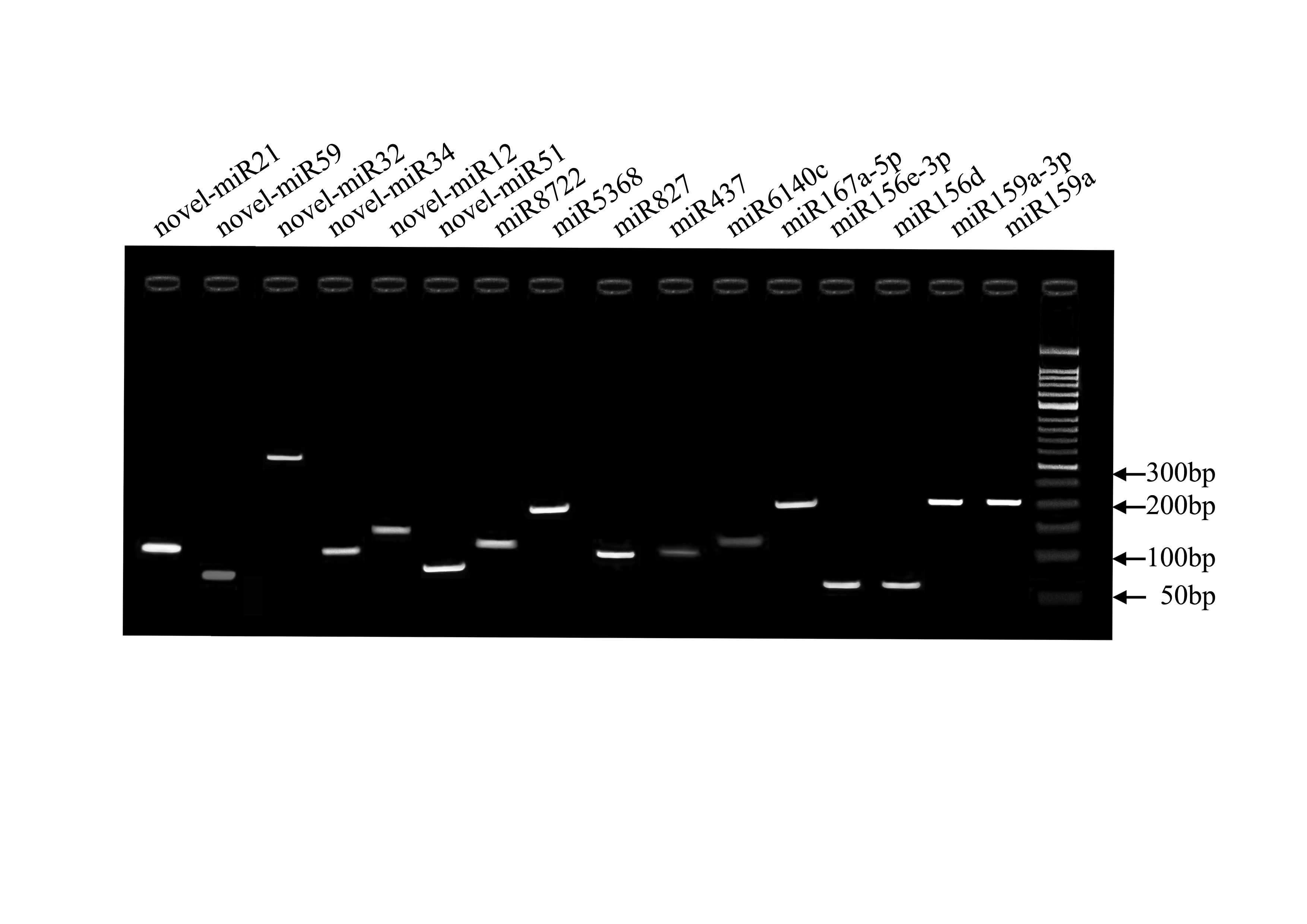


B


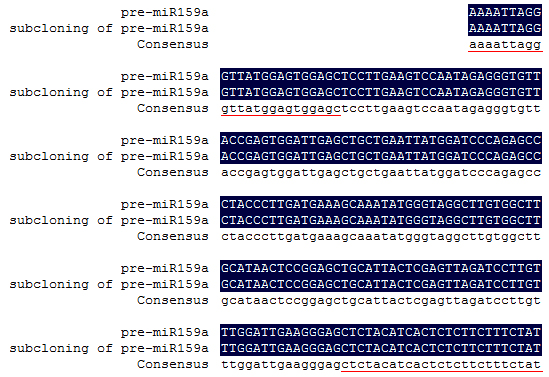


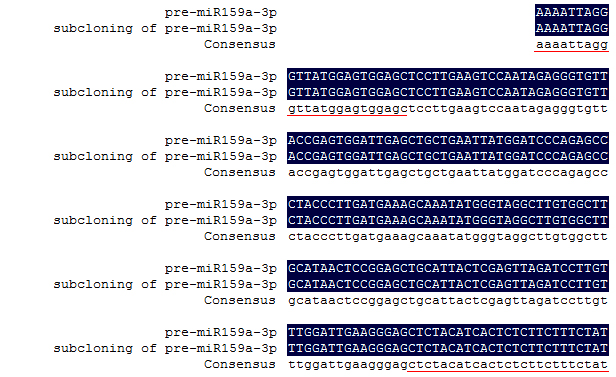


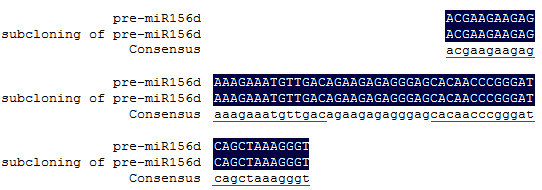


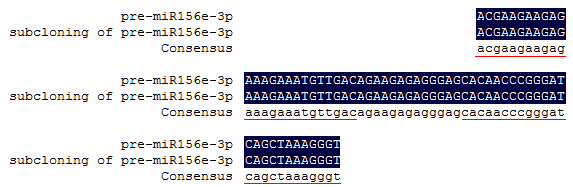


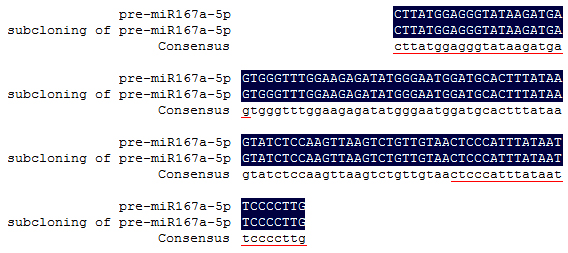


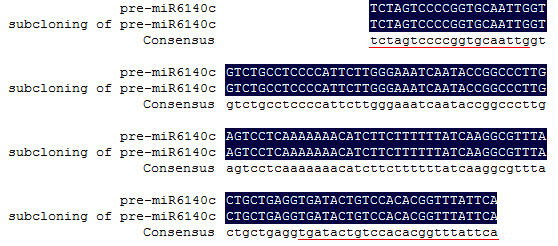


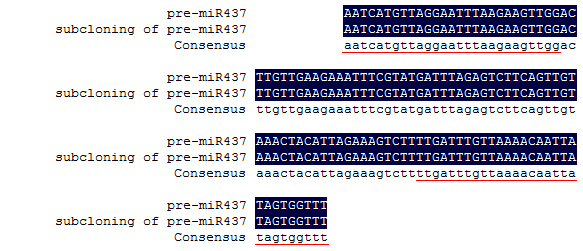


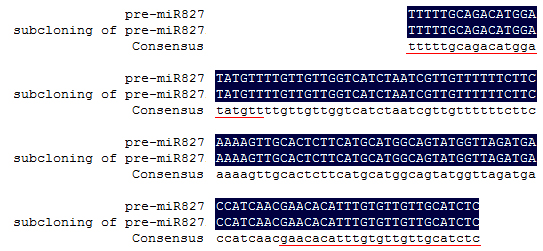


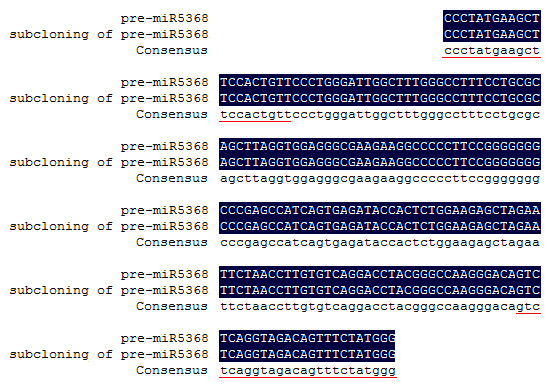


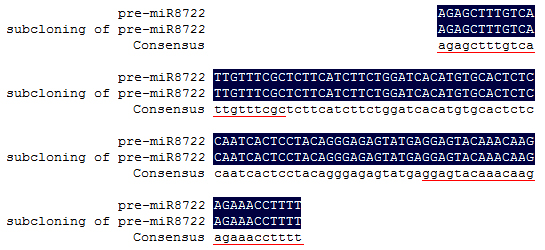


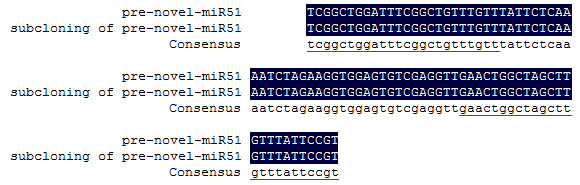


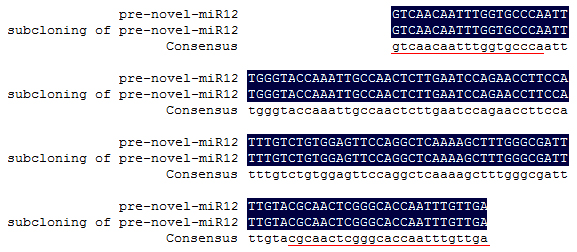


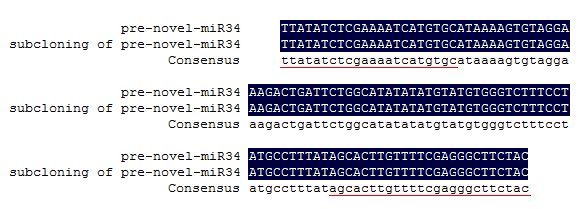


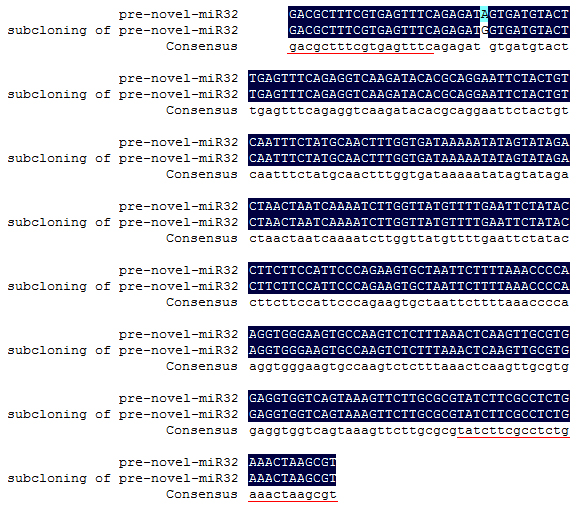


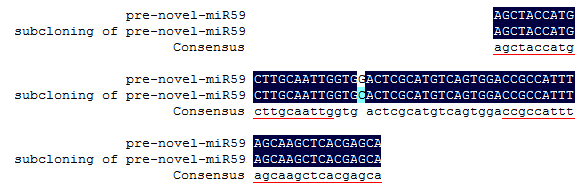


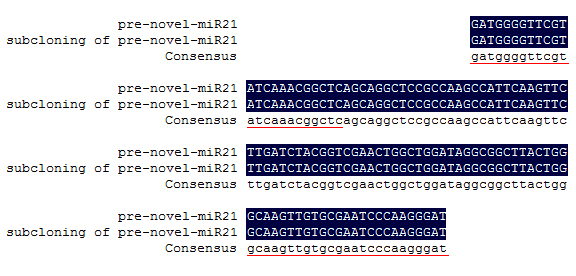


**Supplemental Figure S4.** **The pre-miRNA sequences by PCR amplification (A) and sequencing (B).** Red underline indicated the gene-speciﬁc primers sequence for subcloning of pre-miRNA sequences.

**Supplemental Table S1 Summary of data cleaning produced by small RNA sequencing in two libraries from red outer-petal and yellow inner-petal**.

| type | outer-petal | | inner-petal | |
| --- | --- | --- | --- | --- |
|  | count | percent (%) | count | percent (%) |
| total reads | 12,637,298 |  | 12,353,097 |  |
| high quality | 12,585,513 | 100 | 12,210,957 | 100 |
| 3′ adapter null | 95,569 | 0.76 | 141,273 | 1.16 |
| insert null | 5,740 | 0.05 | 6,037 | 0.05 |
| 5′ adapter contaminants | 15,434 | 0.12 | 15,372 | 0.13 |
| smaller than 18 nt | 127,643 | 1.01 | 128,751 | 1.05 |
| poly A | 1,050 | 0.01 | 846 | 0.01 |
| clean reads | 12,340,077 | 98.05 | 11,918,678 | 97.61 |

**Supplemental Table S2 Summary of data cleaning produced by small RNA sequencing in the other two replicates of red outer-petal and yellow inner-petal.**

| type | outer-petal 2 | | outer-petal 2 | | inner-petal 3 | | inner-petal 3 | |
| --- | --- | --- | --- | --- | --- | --- | --- | --- |
|  | count | percent (%) | count | percent (%) | count | percent (%) | count | percent (%) |
| total reads | 12,324,824 |  | 12,429,479 |  | 12,053,490 |  | 12,095,973 |  |
| high quality | 12,275,768 | 100 | 12,281,248 | 100 | 12,005,214 | 100 | 12,048,066 | 100 |
| 3′ adapter null | 129,118 | 1.05 | 171,700 | 1.40 | 101,452 | 0.85 | 93,689 | 0.78 |
| insert null | 6,096 | 0.05 | 6,124 | 0.05 | 6,196 | 0.05 | 8,277 | 0.07 |
| 5′ adapter contaminants | 17,518 | 0.14 | 19,669 | 0.16 | 19,142 | 0.16 | 15,095 | 0.13 |
| smaller than 18 nt | 214,805 | 1.75 | 227,241 | 1.85 | 137,855 | 1.15 | 182,480 | 1.51 |
| poly A | 822 | 0.01 | 742 | 0.01 | 587 | 0.00 | 706 | 0.01 |
| clean reads | 11,907,409 | 97 | 11,855,772 | 96.54 | 11,739,982 | 97.79 | 11,747,819 | 97.51 |

**Supplemental Table S3 GO functional annotation of target genes from conserved miRNAs.**

| Ontoloty | GO term | Number of target genes |
| --- | --- | --- |
| Biological process | Biological adhesion | 1 |
|  | Biological regulation | 31 |
|  | Cellular component organization or biogenesis | 18 |
|  | Cellular process | 79 |
|  | Developmental process | 24 |
|  | Growth | 3 |
|  | Immune system process | 2 |
|  | Localization | 20 |
|  | Metabolic process | 77 |
|  | Multi-organism process | 6 |
|  | Multicellular organismal process | 21 |
|  | Negative regulation of biological process | 12 |
|  | Positive regulation of biological process | 8 |
|  | Regulation of biological process | 28 |
|  | Reproduction | 2 |
|  | Reproductive process | 9 |
|  | Response to stimulus | 32 |
|  | Rhythmic process | 1 |
|  | signaling | 7 |
|  | Single-organism process | 58 |
| Cellular component | Cell | 82 |
|  | Cell junction | 3 |
|  | Cell part | 82 |
|  | Extracellular region | 3 |
|  | Macromolecular complex | 14 |
|  | Membrane | 34 |
|  | Membrane part | 13 |
|  | Membrane-enclosed lumen | 7 |
|  | Nucleoid | 2 |
|  | Organelle | 65 |
|  | Organelle part | 20 |
|  | Symplast | 3 |
| Molecular function | Antioxidant activity | 1 |
|  | Binding | 57 |
|  | Catalytic activity | 65 |
|  | Electron carrier activity | 2 |
|  | Enzyme regulator activity | 2 |
|  | Transporter activity | 9 |

**Supplemental Table S4 GO functional annotation of target genes from novel miRNAs.**

| Ontoloty | GO term | Number of target genes |
| --- | --- | --- |
| Biological process | Cellular component organization or biogenesis | 3 |
|  | Cellular process | 6 |
|  | Developmental process | 1 |
|  | Localization | 1 |
|  | Metabolic process | 5 |
|  | Multi-organism process | 1 |
|  | Multicellular organismal process | 1 |
|  | Reproduction | 2 |
|  | Reproductive process | 1 |
|  | Response to stimulus | 4 |
|  | Single-organism process | 6 |
| Cellular component | Cell | 9 |
|  | Cell part | 9 |
|  | Extracellular region | 1 |
|  | Macromolecular complex | 1 |
|  | Membrane | 2 |
|  | Organelle | 7 |
|  | Organelle part | 2 |
| Molecular function | Binding | 5 |
|  | Catalytic activity | 5 |

| miRNA ID | Fold-change  (Log2 inner-petal/outer-petal) | Target genes | Annotation | Fold-change  (Log2 inner-petal/outer-petal) |
| --- | --- | --- | --- | --- |
| miR156d | -1.295655713 | Unigene5530 | Squamosa promoter-binding protein-like | 1.1126 |
| miR156e-3p | -3.129029282 | Unigene5530 | Squamosa promoter-binding protein-like | 1.1126 |
|  |  | Unigene10411 | Squamosa promoter-binding protein-like 1 | 0.4008 |
| miR828a | -5.801582652 | Unigene1000 | MYB114 transcription factor | 0.8716 |
|  |  | Unigene22773 | WER-like transcription factor | -0.1285 |
| miR2616 | -6.226888487 | Unigene23856 | Flavonoid 3-*O*-glucosyltransferase | -0.255 |
|  |  | CL7527.Contig2 | Unknown | -0.2659 |
|  |  | CL7527.Contig3 | Unknown | 0.4122 |
| novel-miR25 | -1.657241965 | Unigene35437 | Flavonoid 3-*O*-glucosyltransferase 7-like | 6.3006 |
|  |  | Unigene11591 | Putative DNA repair protein RAD23-1-like | -0.5877 |
|  |  | Unigene15261 | Kanadaptin-like | 0.1326 |
|  |  | Unigene26600 | Unknown | 1.5821 |
|  |  | CL6511.Contig2 | Unknown | -2.81 |

**Supplemental Table S5 Candidate miRNAs and their corresponding target genes.**

**Supplemental Table S6 Gene-speciﬁc primers sequence for subcloning of pre-miRNA sequences**.

| miRNA | Forward primer (5'-3') | Reverse primer (5'-3') |
| --- | --- | --- |
| miR156d | ACGAAGAAGAGAAAGAAATGTTGAC | ACCCTTTAGCTGATCCCGGGTTGTG |
| miR156e-3p | ACGAAGAAGAGAAAGAAATGTTGAC | ACCCTTTAGCTGATCCCGGGTTGTG |
| miR159a | AAAATTAGGGTTATGGAGTGGAGC | ATAGAAAGAAGAGAGTGATGTAGAG |
| miR159a-3p | AAAATTAGGGTTATGGAGTGGAGC | ATAGAAAGAAGAGAGTGATGTAGAG |
| miR167a-5p | CTTATGGAGGGTATAAGATGAG | CAAGGGGAATTATAAATGGGAG |
| miR437 | AATCATGTTAGGAATTTAAGAAGTTGG | AAACCACTATAATTGTTTTAACAAATCAA |
| miR827 | TTTTTGCAGACATGGATATGTT | GAGATGCAACAACACAAATGTGTTC |
| miR5368 | CCCTATGAAGCTTCCACTGTT | CCCATAGAAACTGTCTACCTGAGAC |
| miR6140c | TCTAGTCCCCGGTGCAATTG | TGAATAAACCGTGTGGACAGTATCA |
| miR8722 | AGAGCTTTGTCATTGTTTCGC | AAAAGGTTTCTCTTGTTTGTACTCC |
| novel-miR12 | GTCAACAATTTGGTGCCCA | TCAACAAATTGGTGCCCGAGTTGCG |
| novel-miR21 | GATGGGGTTCGTATCAAACGGCTC | ATCCCTTGGGATTCGCACAACTTGC |
| novel-miR32 | GACGCTTTCGTGAGTTTC | ACGCTTAGTTTCAGAGGCGAAGATA |
| novel-miR34 | TTATATCTCGAAAATCATGTGC | GTAGAAGCCCTCGAAAACAAGTGCT |
| novel-miR51 | TCGGCTGGATTTCGGCTGTTTGTT | ACGGAATAAACAAGCTAGCCAGTTC |
| novel-miR59 | AGCTACCATGCTTGCAATTG | TGCTCGTGAGCTTGCTAAATGGCGG |

**Supplemental Table S7 Gene-speciﬁc primers sequence for reverse-transcription of miRNAs.**

| miRNA | Primer (5'-3') |
| --- | --- |
| miR156e-3p | GTCGTATCCAGTGCAGGGTCCGAGGTATTCGCACTGGATACGACGATGAC |
| miR156d | GTCGTATCCAGTGCAGGGTCCGAGGTATTCGCACTGGATACGACGTGCTC |
| miR2616 | GTCGTATCCAGTGCAGGGTCCGAGGTATTCGCACTGGATACGACCTCGCC |
| miR828a | GTCGTATCCAGTGCAGGGTCCGAGGTATTCGCACTGGATACGACTGGAAT |
| Novel-miR25 | GTCGTATCCAGTGCAGGGTCCGAGGTATTCGCACTGGATACGACGATTAG |

**Supplemental Table S8 Gene-speciﬁc primers sequence for detection by Q-PCR**.

| Gene | Forward primer (5'-3') | Reverse primer (5'-3') |
| --- | --- | --- |
| *Actin* | GCAGTGTTCCCCAGTATT | TCTTTTCCATGTCATCCC |
| miR156e-3p | CGCGCTCTCTCTTCGTCT | GTGCAGGGTCCGAGGT |
| miR156d | GCGCTTGACAGAAGAGAGG | GTGCAGGGTCCGAGGT |
| miR2616 | GCATTGTGGTCTTGGATTCG | GTGCAGGGTCCGAGGT |
| miR828a | GCGCTCTTGCTCAAATGAGT | GTGCAGGGTCCGAGGT |
| novel-miR25 | GCGCTTTTGTTGTTGGTCAT | GTGCAGGGTCCGAGGT |
| Unigene5530 | GCAGCAGGTTCCATTCAT | TCTGGCTGAGGCTTTCTT |
| Unigene10411 | AAGGATTCAGTGGAGGGA | GACAGAAACGCTGTTGGA |
| Unigene1000 | AAACAGGTGGTCTTTGATTG | CAGGCGTGGATTCTTCTC |
| Unigene23856 | CTCCTTACCATCATCCGCTAC | GGCACGCCATCAGAAACA |
| Unigene35437 | CGACTCCAAGAAACCCAA | CTTTACTCACAACCCAGAT |
